# A Quack Recipe

**Published:** 1864-01

**Authors:** 


A QUACK RECIPE.—The following recipe, which is now being sold by a celebrated traveling Doctor, was purchased by E. Booth, of Crestline, O., and sent to the Buffalo Medical and Surgical Journal, with the request that it be published for the good of the profession and humanity in general:

"Medicine for to distract the Reumatism, Pains and toothake, Bachake and boils, strains for man & horses & many more sores: Take one quart of good Old Rye Whiskey & add in the bottle One oz of campfire, six pots of Red pepper, six cents worth of gloves, 5 cents worth of cinnamund, 3 cents worth of shaven sope, One table spoonful of salt. Add them all in the bottle together & set the bottle in the sun 9 days before using & where the pain is grease it well Evening and Morning. Dont give up soon. Shak the bottle 2 or 3 times a day. 50 cents for a reseat. It is a sure cure."

